# Longitudinal analysis of 20 Years of external quality assurance schemes for PCR/NAAT-based bacterial genome detection in diagnostic testing

**DOI:** 10.3389/fmolb.2024.1373114

**Published:** 2024-03-27

**Authors:** Marcel Kremser, Nathalie Weiss, Anne Kaufmann-Stoeck, Laura Vierbaum, Silke Kappler, Ingo Schellenberg, Andreas Hiergeist, Volker Fingerle, Michael Baier, Udo Reischl

**Affiliations:** ^1^ INSTAND e.V., Society for Promoting Quality Assurance in Medical Laboratories, Duesseldorf, Germany; ^2^ Institute of Bioanalytical Sciences (IBAS), Center of Life Sciences, Anhalt University of Applied Sciences, Bernburg, Germany; ^3^ Institute of Clinical Microbiology and Hygiene, University Hospital Regensburg, Regensburg, Germany; ^4^ Bavarian Health and Food Safety Authority, Oberschleißheim, Germany; ^5^ Institute of Medical Microbiology, University Hospital Jena, Jena, Germany

**Keywords:** EQA, *Borrelia burgdorferi*, MRSA, INSTAND, diagnostic molecular pathology

## Abstract

**Background:**

Quality control (QC), quality assurance, and standardization are crucial for modern diagnostic testing in the field of medical microbiology. The need for efficient QC to ensure accurate laboratory results, treatment, and infection prevention has led to significant efforts in standardizing assay reagents and workflows. External quality assessment (EQA) schemes, like those offered by INSTAND, play a vital role in evaluating in-house and commercial routine diagnostic assays, regarded as mandatory by national and global guidelines. The recent impact of polymerase chain reaction/nucleic acid amplification technology (PCR/NAAT) assays in medical microbiology requires that high-performing assays be distinguished from inadequately performing ones, especially those made by inexperienced suppliers.

**Objectives:**

The study assesses the evolving diagnostic performance trends over 2 decades for the detection of EHEC/STEC, *Borrelia* (*B.) burgdorferi*, and MRSA/cMRSA. It explores the historical context of assay utilization, participant engagement, and rates of correct results in EQA schemes. The research seeks to identify patterns in assay preferences, participant proficiency, and the challenges encountered in detecting emerging variants or clinical strains.

**Results:**

The study highlights the decline in in-house PCR assay usage, the emergence of new diagnostic challenges, and educational aspects within EQA schemes. Specific examples, such as the inclusion, in certain EQA surveys, of EHEC strains carrying *stx*-2f or *B. miyamotoi*, highlight the role of EQAs in increasing awareness and diagnostic capabilities. Advancements in MRSA detection, especially through the adoption of commercial assays, demonstrate the impact that technology evolution has had on diagnostic performance.

**Conclusion:**

Achieving excellence in diagnostic molecular microbiology involves a multifaceted approach, including well-evaluated assays, careful instrumentation selection, and structured training programs. EQA schemes contribute significantly to this pursuit by providing insights into the evolving diagnostic landscape and identifying areas for improvement in the diagnostic workflow as well as in PCR/NAAT assay design.

## 1 Introduction

Quality control (QC), quality assurance and standardization are among the most important prerequisites for modern diagnostic testing in medical microbiology and infectious diseases. Next to the use of well-evaluated assay concepts, the establishment and maintenance of efficient QCs are vital to ensuring the accuracy of laboratory results. This enables accurate patient identification and treatment as well as effective infection prevention (Badrick, 2021). Over the past decades, huge efforts have been made in standardizing assay reagents, creating diagnostic workflows, and incorporating internal controls with the aim of achieving results with the highest level of accuracy and reliability. External quality assessment (EQA) schemes are a crucial component in the reliable performance of routine diagnostic assays for pathogens or genetically encoded pathogenicity factors in medical microbiology and infectious diseases (Laudus et al., 2022). The value of regular participation is beyond dispute and hence mandatory in the official guidelines and regulations of most countries worldwide (De la Salle et al., 2017).

In the wake of the recent global pandemic, the commercial market has been flooded by many new assay concepts and instruments based on polymerase chain reaction/nucleic acid amplification technology (PCR/NAAT). These range from manual to semi- or fully automated systems and closed assay cartridges. Within this landscape, it is important to be able to distinguish between high-performing assays and assays from inexperienced suppliers that have inadequate analytical performance levels in real-world clinical settings.

Hence, there is a need to identify the many assays or test kits, supplied by inexperienced manufacturers, with inadequate performance in routine testing.

One of the significant challenges in diagnostic microbiology is the accurate detection of various pathogens, including bacteria and fungi. These microorganisms pose diverse challenges due to factors such as their genetic variability, rapid evolution, and the emergence of antimicrobial resistance or certain virulence factors. Accurate diagnosis is critical to reducing the spread of infectious diseases, optimizing patient management, and preventing adverse outcomes. Misdiagnosis or delayed diagnosis can lead to inappropriate treatment, disease progression, and potential transmission to others (Fournier et al., 2013). Therefore, the importance of precise and timely diagnosis cannot be overstated, especially in the context of these pathogens with significant nosocomial and/or public health implications.

INSTAND EQA schemes cover a broad range of relevant bacterial and fungal pathogens and are designed to identify and pinpoint potential weaknesses of certain PCR/NAAT assay concepts. Continuous participation not only serves as a benchmarking tool, as it is a way to obtain official certificates, it also has an educational effect. Retrospective studies reveal an improvement in laboratory performance among laboratories that regularly participate in EQA schemes, highlighting the educational role of EQAs (Keppens et al., 2018; Keppens et al. 2019; Keppens et al. 2021). The random inclusion of so-called “educative samples” among the selected target organisms reflects a primary commitment to the ongoing advances within the field of diagnostic medical microbiology and, consequently raises awareness of participants to new, emerging, or interesting genetic variants or clinical strains.

The INSTAND EQA project for the detection of bacterial DNA started in 2003 with biannual distributions of sample sets for *Chlamydia (C.) trachomatis*, *Neisseria gonorrhoeae*, *Bordetella pertussis*, *Helicobacter pylori*, enterohemorrhagic *Escherichia (E.) coli/*shigatoxigenic *Escherichia coli* (EHEC/STEC), *B. burgdorferi*, *Legionella pneumoniae*, *Salmonella enterica* and *Listeria* species (spp.). However, with the widespread adoption of PCR/NAAT-based assays in diagnostic medical microbiology, the EQA program has progressively broadened its spectrum and continues to grow.

The expanded EQA scheme now includes surveys for Methicillin-resistant *Staphylococcus aureus/*community acquired Methicillin-resistant *S. aureus* (MRSA/cMRSA), *C. pneumoniae*, *Mycoplasma pneumoniae*, *Bacillus anthracis*, *Coxiella burnetii*, *Francisella tularensis*, *Brucella* spp., Carbapenemases genes, toxinogenic *Clostridium difficile*, Vancomycin-resistant *Enterococci* (VRE), *Pneumocystis jirovecii*, and a comprehensive panel of bacterial urogenital pathogens that address recent multiplex PCR assay concepts.

Each EQA set comprises four samples containing various concentrations of the target organism as well as related species or *E. coli* cells as negative set members. Despite the great diagnostic potential of PCR testing, the success of each of its analytical applications is highly dependent on the reliability of the clinical samples containing nucleic acids for amplification. While EQA schemes may not perfectly mimic the range of different PCR inhibitors that are complicating real-world sample analysis (e.g., false-negative results or insufficient lower limits of detection) (Vesper et al., 2007), the proprietary matrix of lyophilized samples, composed of proteins, salts, and a significant number of human cells, enables the semiquantitative detection of human gene segments. This makes them valuable for use as purification, extraction, and/or inhibition controls.

While advancements in diagnostic technologies have undoubtedly improved the accuracy and efficiency of pathogen detection, there remain gaps in our understanding of the evolving trends in diagnostic methods and their performance over time. Existing literature highlights the transition from traditional culture-based methods to advanced molecular techniques like PCR/NAAT for rapid microbial identification and specific characterization (Weile and Knabbe, 2009; Das et al., 2017). However, there is limited research investigating the longitudinal trends in diagnostic accuracy and performance of these molecular assays, especially concerning their adaptation to changing clinical needs and emerging infectious threats. The study is the first to address this question by performing a longitudinal analysis over 20 years of EQAs for PCR/NAAT-based bacterial genome detection of EHEC/STEC, *B*. *burgdorferi* and for MRSA/cMRSA. Through this analysis, the study seeks to provide accessory insights into the evolving landscape of diagnostic testing, identifying patterns, challenges, and improvements in performance, thereby contributing valuable knowledge to the field.

## 2 Materials and methods

### 2.1 EQA procedure

The INSTAND EQA schemes for bacterial genome detection of EHEC/STEC (EQA 534), *B. burgdorferi* (EQA 535), and MRSA or cMRSA (EQA 539) were conducted globally twice a year (surveys in May and November) and contained four different samples per survey (4 × 0.3 mL). Detailed sample properties and compositions of microorganisms can be found in Supplementary Table S1. The stability and homogeneity of the EQA samples were assessed according to DIN EN ISO/IEC 17043:2023 standards (ISO/IEC17043:2023, 2023). To process the samples, the laboratories had to centrifuge the vials containing lyophilized material. The material was then reconstituted in 300 µL of sterile water (PCR grade) and incubated at room temperature for 20 min on an orbital shaker and/or with occasional vortexing. This resulted in suspensions comparable to native clinical specimens. 100 μL aliquots had to be processed using typical protocols for DNA extraction and PCR/NAAT assays established in the laboratories’ routine diagnostic setting. Participating laboratories were tasked with determining qualitative outcomes (positive, negative, questionable) and were asked to submit their findings to the INSTAND “RV-Online” web portal (http://rv-online.instandev.de). Alongside the qualitative results, participants had to submit information on the methods used for DNA extraction and amplification, and specified which commercial kits were used or whether an in-house PCR assay (lab-developed test, LDT) was used. For all three EQA schemes, successful certification required an accurate determination of three out of four samples, as stipulated by the current guidelines of the German Medical Association (RiliBÄK) (Bundesärztekammer, 2023).

### 2.2 Data analysis and statistics

The EQA results for EHEC/STEC, *B. burgdorferi*, and MRSA or cMRSA were analyzed in a manufacturer-specific manner across surveys performed between November 2003 and May 2023. The MRSA EQA scheme started in November 2005. A limited number of results (*n* = 2) were reported in November 2007 for the EQA survey detecting EHEC/STEC, making a test-specific analysis statistically less robust. Hence, this survey was excluded from the study. This resulted in 39 surveys for EHEC/STEC, 40 for *B. burgdorferi*, and 36 for MRSA.

For all three pathogens, assay manufacturer collectives with the highest participant counts per survey were represented individually. In the case of EHEC/STEC, the six most common methods were presented, while for *B. burgdorferi* this number was seven, and for MRSA or cMRSA it was nine. The remaining commercial test kits or preconfigured PCR/NAAT assay concepts were combined into the category “other.” Bar charts were used to illustrate the distribution of participating assay-specific laboratories over time for EHEC/STEC, *B. burgdorferi*, and MRSA or cMRSA. In order to discern potential trends over the years, percentages of correct results per date and sample were graphically depicted for each EQA scheme, with symbols indicating specific events. These events included the utilization of clinical variants, very low concentrations, and possible cross-contamination. A sample was considered correct when the presence or absence of the target microorganism was detected accurately. We analyzed the data based on the percentage of correctly identified samples in each survey per sample. Basic statistical analyses were performed using JMP 17.0.0 from SAS Institute (Cary, North Carolina, USA).

Overlay images were created using the Gnu image manipulator software 2.10.34.

## 3 Results

**FIGURE 1:**
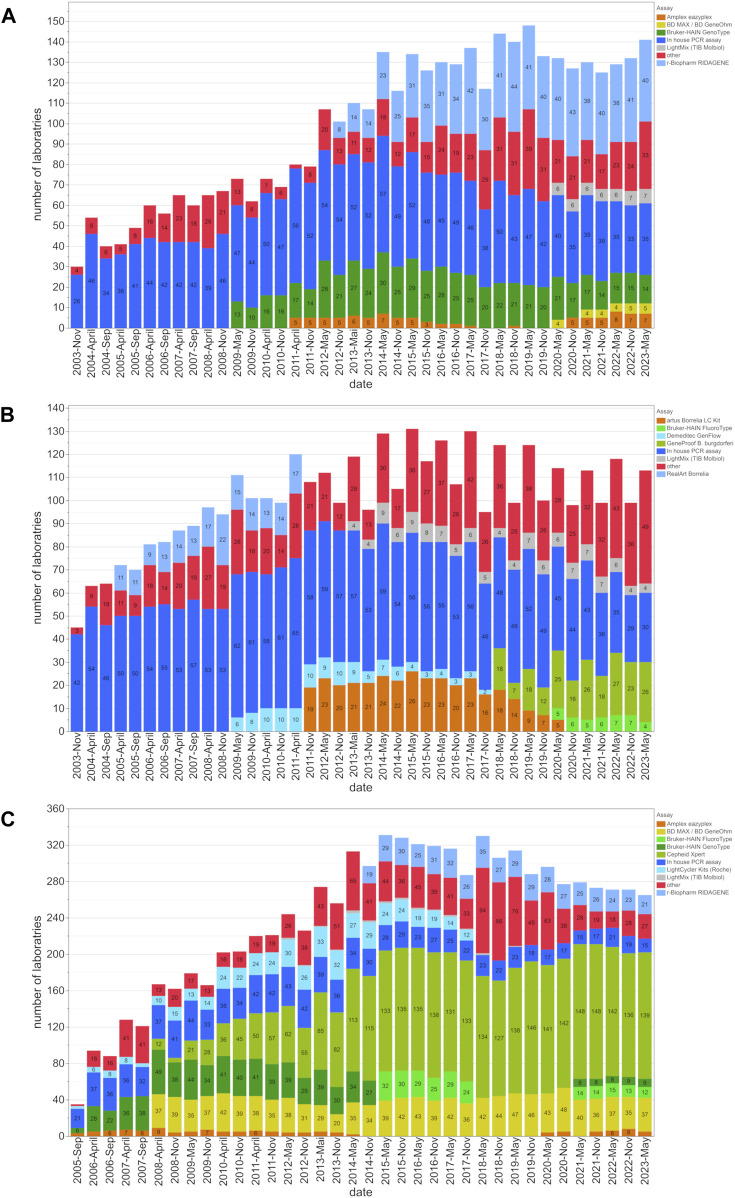
Assay distribution and number of participating laboratories from 2003 to 2023. This figure shows the distribution of assay utilization among participating laboratories and the changes in the utilization of these assays for the **(A)** EHEC/STEC, **(B)** *B. burgdorferi*, and **(C)** MRSA/cMRSA EQA schemes. The number of laboratories employing a certain assay type is indicated within the bars for each EQA scheme.

**FIGURE 2:**
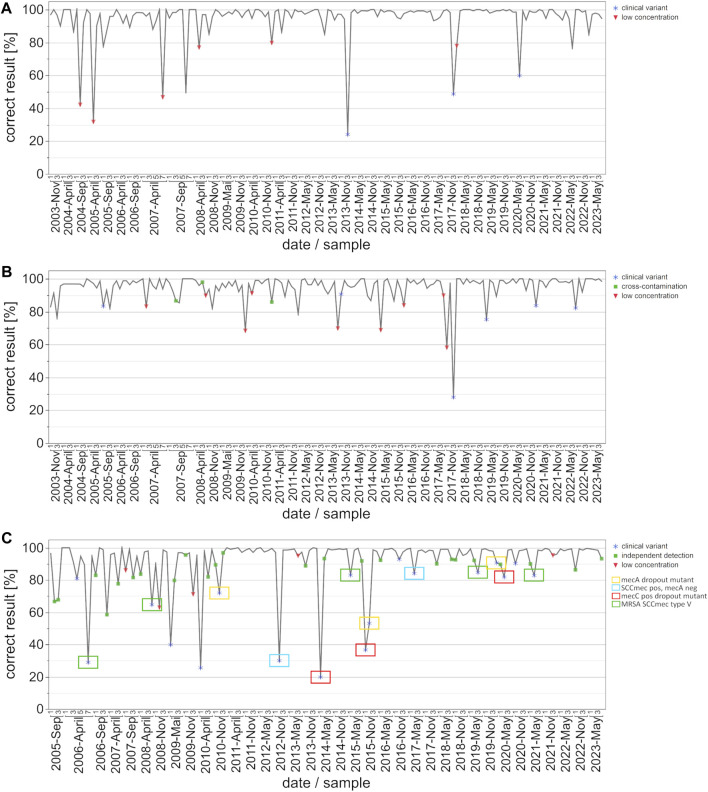
Development of correct results for the detection auf EHEC/STEC **(A)**, *B. burgdorferi* **(B)** and MRSA/cMRSA **(C)** from 2003 to 2023 with emphasis on key events/special sample composition. The data points on the graph represent the percentage of correct results per survey and date. Key events are defined as clinical variants (blue star), low concentrations of the respective target organisms (red triangle), and potential cross-contamination or independent detection (green square). The clinical variants were subdivided into four categories for MRSA: *mec*A dropout mutant (yellow box), SCC*mec* cassette positive but *mec*A negative MSSA (blue box), *mec*C positive MRSA variant (red box), and MRSA with an SCC*mec* type V cassette (green box).

This study evaluated the inter-laboratory detection quality for EHEC/STEC and *B*. *burgdorferi* from November 2003 to May 2023, and for MRSA/cMRSA from November 2005 to May 2023. In order to identify the evolving trends, we analyzed up to forty EQA surveys during this period, looking at the number of participating laboratories, assay distribution (Figure 1), and rates of correct results (Figure 2).

The number of EQA participants for EHEC/STEC, *B*. *burgdorferi,* and MRSA increased from 30, 45, and 35, to 148, 131, and 331 respectively. At the beginning, 60% (MRSA), 87% (EHEC/STEC), and 93% (*B*. *burgdorferi*) of laboratories used in-house PCR assays. However, these percentages gradually declined over the years as commercially available assays gained prominence. By May 2023, the utilization of in-house PCR assays dropped to 24.8% (EHEC/STEC), 26.5% (*B*. *burgdorferi*), and 5.7% (MRSA) (Figure 1).

In order to analyze the progression of pass rates and testing quality, we graphically illustrated correct results [%] per date and sample, with symbols indicating either clinical variants, low pathogen concentrations, or cross-contamination (Figure 2). In the case of EHEC/STEC detection, correct results exceeded 85%, with instances of lower percentages typically corresponding to samples involving very low target organism concentrations or special clinical variants (Figure 2A). The only clinically relevant variants during the observation period were *stx*-2f and *eae* positive; the rates of correct results for these variants increased from about 24% to 60%.

Similar to the EHEC/STEC findings, the rate of correct results for *B*. *burgdorferi* consistently surpassed 90%, with instances of lower percentages often linked to very low pathogen concentrations, clinical variants, or possible cross-contamination (Figure 2B).

For the MRSA and cMRSA EQA schemes, instances with fewer correct results were notably associated with clinical variants and very low pathogen concentrations (Figure 2C). Additionally, green squares represent methicillin-susceptible *S. aureus* (MSSA) + coagulase-negative staphylococci (CONS) samples, for which “questionable” results from participants were included as correctly positive. This indicates the use of separate assays for detecting the mecA gene and a *S. aureus* species marker gene. Worth noting is the classification of clinical variants, with a particular focus on the four recurring categories: *mec*A dropout mutant (yellow box), SCC*mec* cassette positive but *mec*A negative MSSA (blue box), *mec*C positive MRSA variant (red box), and MRSA with an SCC*mec* type V cassette (green box). Rates of correct results for these four specific clinical variants improved over the years from under 50% to approximately 90%. The overall rate of correct results for MRSA and cMRSA consistently surpassed 90%, with instances of lower percentages often linked to very low pathogen concentrations, clinical variants, or the use of separate assays for detecting the mecA gene and a *S. aureus* species marker gene.

## 4 Discussion

Statistical analyses of EQA schemes, deliberately designed with highly diverse sample compositions in each survey, pose a complex challenge. These schemes lack a simple standard or comparator across different sample sets. Nonetheless, examining the percentage of correct results over nearly 2 decades offers valuable insights into assay standardization, coverage of variant bacterial pathogen strains, the analytical sensitivity for detecting relatively low concentrations of the respective target organisms, and the analytical specificity for distinguishing between the pathogen and the less pathogenic or apathogenic strains within a species or genus.

One illustrative example is the inclusion of clinically relevant variants of common pathogens like the Swedish *Chlamydia trachomatis* variant nvCT (Reischl et al., 2009). This new *C. trachomatis* variant was first identified in 2006 in the Swedish province of Halland and is characterized by a 377-bp deletion in the ORF-1 coding region of the multicopy cryptic plasmid. This region was targeted by both the Roche and Abbott *C. trachomatis* PCR assays available at the time. This nvCT strain was included in INSTAND’s May 2009 EQA survey, in which the 128 participating laboratories used at least twelve different commercial PCR test kits or assays and a broad spectrum of in-house PCR assays. As expected, about 20% of the participants did not detect the *C. trachomatis* nvCT DNA in the sample when using the specific version of the Roche COBAS Amplicor CT/NG or several other, unspecified in-house PCR assays. When the nvCT strain was incorporated into a subsequent survey in May 2010, there was a notable increase in the accurate detection rate. It appears that the laboratories previously experiencing issues, as well as commercial PCR assay development teams, learned from this experience and subsequently redesigned their PCR assays to cover this variant strain (Reischl et al., 2010).

The examples of EHEC, *B. burgdorferi* and MRSA selected for this study emphasize the growing trend in utilizing prefabricated commercial PCR kits or closed cartridge-based PCR concepts (Figure 1). Many diagnostic laboratories still rely on established in-house or lab-developed tests (LDT) for the PCR/NAAT-based detection of the Shiga toxin-producing *E. coli* (EHEC/STEC). Although their prevalence slightly diminished around 2011 with the widespread availability of commercial PCR kits, in-house PCR assays, as shown in Figure 2A, continue to demonstrate high diagnostic accuracy and compete with commercial kits from various suppliers.

INSTAND’s EQA scheme for the PCR/NAAT-based detection of EHEC/STEC (EQA 534) usually covers the various Shiga toxin genes and the putative accessory virulence marker genes of typical EHEC strains that occur around the world. The popular target genes include Shiga toxin gene variants *stx*-1, *stx*-1c, *stx*-2, *stx-*2c, *stx*-2d and *stx*-2e, as well as *eae*A (intimin) and E-*hly*A (enterohemolysin).

In 2000, a new Shiga toxin two variant (*stx*-2f) was identified in an *E. coli* strain isolated from pigeons (Schmidt et al., 2000). This observation enlarged the pool of *stx*-2 gene variants of human-pathogenic EHEC strains (Sonntag et al., 2005). It should be noted that the *stx*-2f encoding gene is quite distinct from other Shiga toxin gene variants at the nucleotide sequence level. This makes coverage by a common primer pair that targets conserved regions of *stx*-2, *stx*-2c, *stx*-2d, or *stx*-2e challenging. Consequently, modified or adapted assay designs require additional primer pairs and detection probes, complicating the composition of the PCR assay. Composition and subsequent comprehensive clinical re-validation of these assays may be needed.

The EHEC strain carrying *stx*-2f was first included in EQA 534 in November 2013. Similar to the situation with the aforementioned *C. trachomatis* variant, about 80% of the participants failed to detect the Shiga toxin gene variant in the sample when using various commercial test kits or other, unspecified in-house PCR assays. When the same strain was present in November 2017, the rate of correct detection increased to around 50% (58 out of 113 participants). By May 2020, this percentage had risen to around 60% (79 out of 132 participants), indicating the increased availability and use of re-designed commercial or in-house PCR assay concepts over the past decade. This situation is also nicely illustrated in the overall correct results depicted in Figure 2A, where the three outliers in November 2013 November 2017, and May 2020 correspond to the presence of EHEC strains carrying the *stx*-2f gene. Once again, this emphasizes the overall diagnostic advantages of incorporating such emerging or atypical strains of bacterial pathogens for educational purposes. It also raises awareness among colleagues in the fields of diagnostic microbiology and PCR/NAAT assay development of the rise of Shiga toxin variants in the EHEC circulating in animal and human populations. Moreover, the constellation depicted here represents similar situations in other INSTAND EQA schemes for PCR/NAAT-based detection of bacterial or fungal pathogens.

The PCR/NAAT-based detection of *B. burgdorferi* DNA is historically based on a variety of LDTs which evolved as robust and reliable diagnostic tools in the hands of experienced laboratories. With the increasing awareness of borreliosis as an emerging disease, several commercial kits have entered the market, supporting routine laboratories in expanding their diagnostic spectrum for detecting *B. burgdorferi* DNA in various types of clinical samples. Throughout the observed and analyzed time period, both in-house and commercial PCR assays consistently yielded high percentages of correct results, with only occasional interruptions due to samples containing very low numbers of target organisms (Figure 2B).

The *B. burgdorferi* PCR proficiency testing panel is designed for the specific and sensitive detection of *B. burgdorferi* sensu lato (s.l.) DNA, but the positive samples do not necessarily contain suspensions of “prototype” isolates of *B. burgdorferi* sensu stricto. Over the past 2 decades, many EQA surveys contained other *B. burgdorferi* genospecies or related species in individual samples. At least 21 different species are known to belong to the *B. burgdorferi* s.l. complex, which naturally present genetic differences in commonly used target genes. As part of our *B. burgdorferi* scheme, the May 2015 survey contained, in addition to three samples positive for the *B. burgdorferi* s.l. species, one sample with *B*. *miyamotoi* to challenge analytical specificities of PCR/NAAT assays used in the field. This species was first described in Japan in 1994. It belongs to the relapsing fever group of spirochetes but is transmitted by the same Ixodes ticks as *B. burgdorferi* s.l. in the United States, Asia and Europe. The *B*. *miyamotoi* sample was classified as false-positive by 36 of the 128 participating laboratories when certain commercial test kits or in-house PCR assays were used. A similar situation was observed in one sample of *B*. *hispanica* in the November 2020 survey. *B. hispanica* is not a member of the *B. burgdorferi* s.l. complex, but like *B*. *duttonii*, it is one of the causative agents for tick-borne relapsing fever that is present mainly in Spain and Northern Africa. This species is still extremely rare in Europe and of particular diagnostic importance for travelers with febrile illnesses. While the remaining 3 *B. burgdorferi* s.l. positive or negative samples in this particular survey were almost all correctly reported by the 98 participating laboratories, about 15% reported a false-positive result for *B. hispanica* organisms (Reischl et al., 2021). When sample sets contain analytical challenges in good faith and with an educative purpose, it is common practice in the supplementary documentation to encourage participants who obtained false-negative or false-positive results to re-evaluate their assay’s analytical specificity and/or sensitivity. All in all, the inclusion of educative samples in conjunction with a corresponding scientific discussion is very well received by the participants.

MRSA detection improved significantly over the 20-year period with the broader introduction of commercial PCR assays and kits (primarily based on the detection of SCC*mec* cassettes) around the year 2010 (Figure 2C). The ability to discriminate between *mec*A-positive coagulase-negative staphylococcal species, *mec*A-negative *S. aureus* (MSSA), and the most critical *mec*A-positive *S. aureus* strains (MRSA) by covering the SCC*mec* cassette as an additional target is considered a milestone in rapid and reliable screening for MRSA in nasal swabs or other clinical specimens.

A second wave of improvement came with the awareness of *mec*C positive MRSA variants and their subsequent inclusion in some PCR assay concepts in 2017. Since then, an increasing number of commercial or in-house PCR concepts cover the *mec*C gene in addition to the *mec*A gene as potential methicillin-resistance markers in *S. aureus* organisms.

Furthermore, it should be noted that the EQA schemes use clinical isolates rather than classical type strains of a given species. This deliberate choice ensures a more representative assessment of diagnostic proficiency, as clinical isolates better reflect the complexities and variations encountered in real-world scenarios. By incorporating such clinically relevant strains, the EQA schemes aim to more accurately evaluate the ability of laboratories to detect MRSA or other pathogenic bacterial species of clinical relevance under conditions that closely mimic true clinical settings. Supplementary Table S2 provides additional insight into the diverse clinical variants considered in EQA scheme 539, specifically tailored to MRSA/cMRSA.

Over the past 2 decades, the percentage of in-house PCR assays has gradually decreased over the years. By May 2023, the use of in-house PCR assays decreased from 60% (MRSA), 87% (EHEC/STEC), and 93% (*B*. *burgdorferi*) to 24.8% (EHEC/STEC), 26.5% (*B*. *burgdorferi*), and 5.7% (MRSA). While the exact reasons for this shift remain unclear, a plausible explanation could be attributed to Regulation (EU) 2017/746 (IVDR) and its implementation of EU-wide, harmonized requirements for *in vitro* diagnostic medical devices in European healthcare institutions, which took full effect on 26 May 2022 (The European Parliament, 2017). Under the new EU regulation, healthcare institutions in the EU may continue to manufacture and use self-developed diagnostic products, provided they comply with the provisions outlined in Article 5 (5) of the regulation. However, certain requirements under the IVDR have been expanded beyond those of the previous regulations, resulting in increased validation and documentation efforts for medical laboratories (Hoffmuller et al., 2021). Consequently, the IVDR may be responsible for the gradual decline in the use of in-house PCR assays, as laboratories may increasingly switch to commercially available assays on the market that offer a more convenient solution amidst the increased regulatory requirements. In addition, the proliferation of commercial assays on the market provides laboratories with a wider range of options, further incentivizing the adoption of these commercially available assays over those developed in-house.

It is important to note that the overall diagnostic performance of individual laboratories is not solely determined by using “perfect” PCR assays. It also hinges on the careful selection and structured use of instrumentation, as well as the accurate execution of various manual steps throughout the entire workflow, including preanalytical and postanalytical processes.

Throughout the various EQA schemes, evident cross-contamination events during the consecutive steps of sample handling, automated or manual DNA preparation, and preparation of the PCR reaction mixtures mainly occurred when highly positive samples were present in individual sets. Laboratories that obtained such false-positive results due to contamination were clearly encouraged to monitor their individual diagnostic workflow and/or laboratory instrumentation for critical steps and initiate proper optimization measures. In addition to identifying general or specific shortcomings in the analytical sensitivity or specificity of individual PCR/NAAT assays, recognizing cross-contamination risks through regular participation in EQA schemes, and subsequently improving workflows contribute significantly to an overall enhancement of diagnostic quality.

Although this study provides valuable insights into longitudinal trends in diagnostic performance of PCR/NAAT-based bacterial genome detection, it is important to recognize several limitations. First, there may be potential bias in the selection of participants, as laboratories participate in EQA schemes on a voluntary basis, with participation being mandatory only for accredited labs, which may affect the representativeness of the data. In addition, variations in sample composition, including the concentration of target organisms and the presence of interfering substances, may affect assay performance and introduce bias into the results. Furthermore, it is important to note that our study utilized cultured samples rather than primary sample material. This distinction is particularly relevant since swabs often contain lower concentrations of target organisms compared to cultured samples.

Furthermore, the generalizability of our findings to broader scenarios beyond the specific infections analyzed needs to be considered. The dynamics of diagnostic performance observed in the EHEC/STEC, *B. burgdorferi* and MRSA/cMRSA assays may not be directly applicable to other pathogens or testing contexts. Therefore, caution should be exercised when extrapolating these results to other microbial targets or diagnostic settings.

Despite these limitations, our study underscores the importance of continued participation in EQA schemes and highlights the educational role of such programs in improving laboratory performance over time.

## 5 Conclusion

Achieving the highest level of performance in diagnostic molecular microbiology relies on a trifecta of critical elements (I) the use of well-evaluated PCR assay concepts or kits optimized with respect to analytical sensitivity and specificity, (II) a carefully selected and orchestrated instrumentation, and (III) structured programs for ongoing laboratory technician training to assure accurate execution of various manual steps within the workflow. Independent monitoring of the overall diagnostic performance is ultimately accomplished by regular participation in EQA schemes. Successfully meeting EQA requirements leads not only to essential certificates for maintaining the laboratory’s official accreditation status but also to a better diagnostic efficiency that results in improved patient care.

In addition to assessing the diagnostic performance (analytical sensitivity and specificity) of different assays in individual laboratories, a statistical analysis of the results provides an actual snapshot of the technology and the use of commercial or in-house PCR/NAAT assays to detect a given pathogen among the broad and representative cohort of participants.

In essence, EQA schemes are not the sole solution but indeed one of the invaluable tools to preserving diagnostic quality. They provide early insights into potential shortcomings and weaknesses within the often complex and multifaceted diagnostic workflow, and contribute to the pursuit of excellence in diagnostic molecular microbiology.

Looking ahead, future research should continue to monitor diagnostic trends and performance to ensure the continued effectiveness of molecular microbiology diagnostics. In particular, efforts should be directed towards addressing continuous diagnostic challenges, such as the detection of new genetic variants as well as emerging antibiotic resistance genes or new putative virulence factors. In addition, expanding EQA schemes to include a wider range of pathogens and incorporating new technologies, such as next-generation sequencing, could further improve the quality and reliability of diagnostic tests. Collaboration between healthcare providers, regulators and industry stakeholders will be essential to drive innovation and improve patient outcomes in diagnostic medical microbiology.

## Data Availability

The datasets presented in this study can be found in online repositories. The names of the repository/repositories and accession number(s) can be found below: https://homepages.uni-regensburg.de/∼reu24900/INSTAND_e.htm.
